# Therapeutic effect of palmitoylethanolamide in cognitive decline: A systematic review and preliminary meta-analysis of preclinical and clinical evidence

**DOI:** 10.3389/fpsyt.2022.1038122

**Published:** 2022-10-28

**Authors:** Marco Colizzi, Riccardo Bortoletto, Chiara Colli, Enrico Bonomo, Daniele Pagliaro, Elisa Maso, Gianfranco Di Gennaro, Matteo Balestrieri

**Affiliations:** ^1^Unit of Psychiatry, Department of Medicine (DAME), University of Udine, Udine, Italy; ^2^Department of Psychosis Studies, Institute of Psychiatry, Psychology and Neuroscience, King's College London, London, United Kingdom; ^3^Department of Health Sciences, School of Medicine, University of Catanzaro Magna Graecia, Catanzaro, Italy

**Keywords:** neurocognitive disorder, dementia, Alzheimer's disease, Parkinson's disease, cannabinoids, acylethanolamines, immune response

## Abstract

Cognitive decline is believed to be associated with neurodegenerative processes involving excitotoxicity, oxidative damage, inflammation, and microvascular and blood-brain barrier dysfunction. Interestingly, research evidence suggests upregulated synthesis of lipid signaling molecules as an endogenous attempt to contrast such neurodegeneration-related pathophysiological mechanisms, restore homeostatic balance, and prevent further damage. Among these naturally occurring molecules, palmitoylethanolamide (PEA) has been independently associated with neuroprotective and anti-inflammatory properties, raising interest into the possibility that its supplementation might represent a novel therapeutic approach in supporting the body-own regulation of many pathophysiological processes potentially contributing to neurocognitive disorders. Here, we systematically reviewed all human and animal studies examining PEA and its biobehavioral correlates in neurocognitive disorders, finding 33 eligible outputs. Studies conducted in animal models of neurodegeneration indicate that PEA improves neurobehavioral functions, including memory and learning, by reducing oxidative stress and pro-inflammatory and astrocyte marker expression as well as rebalancing glutamatergic transmission. PEA was found to promote neurogenesis, especially in the hippocampus, neuronal viability and survival, and microtubule-associated protein 2 and brain-derived neurotrophic factor expression, while inhibiting mast cell infiltration/degranulation and astrocyte activation. It also demonstrated to mitigate β*-*amyloid-induced astrogliosis, by modulating lipid peroxidation, protein nytrosylation, inducible nitric oxide synthase induction, reactive oxygen species production, caspase3 activation, amyloidogenesis, and tau protein hyperphosphorylation. Such effects were related to PEA ability to indirectly activate cannabinoid receptors and modulate proliferator-activated receptor-α (PPAR-α) activity. Importantly, preclinical evidence suggests that PEA may act as a disease-modifying-drug in the early stage of a neurocognitive disorder, while its protective effect in the frank disorder may be less relevant. Limited human research suggests that PEA supplementation reduces fatigue and cognitive impairment, the latter being also meta-analytically confirmed in 3 eligible studies. PEA improved global executive function, working memory, language deficits, daily living activities, possibly by modulating cortical oscillatory activity and GABAergic transmission. There is currently no established cure for neurocognitive disorders but only treatments to temporarily reduce symptom severity. In the search for compounds able to protect against the pathophysiological mechanisms leading to neurocognitive disorders, PEA may represent a valid therapeutic option to prevent neurodegeneration and support endogenous repair processes against disease progression.

## Introduction

At the neurobiological level, neurocognitive disorders (NCDs) are characterized by reduced neuronal survival and increased neuronal death in the central nervous system (CNS), with a consequent progressive loss of neural function (1–3). Such damages are believed to underpin the cognitive deficits observed at the behavioral level, ranging from mild cognitive impairment to frank NCDs, previously classified as dementias (4). With the progressive increase in life expectancy, the prevalence and incidence of NCDs have dramatically increased, making them leading causes of disability. Specifically, both primary (e.g., Alzheimer's disease, AD) and secondary (e.g., Parkinson's disease, PD) cognitive decline associated with NCDs have increased exponentially during the last years (5), doubling from 1990 to 2016 (6), and are estimated of affecting around 100 million by 2050 (7). This implies a consistent burden for the health-care systems, considering the growing demand for treatments and support services.

Although the pathological mechanisms underlying neurodegenerative diseases are complex and not completely understood, neuroinflammation seems to play a crucial role in the neurodegenerative process (8). The neuroinflammatory response is a protective process that promote neuronal regeneration, however when sustained over time it may lead to neurodegeneration. The main cells involved in this process are microglia and astrocytes and their excessive and prolonged activation has been suggested to produce deleterious effects (9).

Currently, there are no therapeutic agents that effectively counteract the neurodegenerative damage or even slow the progression of these disorders (10). In this context, targeting and modulating neuroinflammation pathways seems to be a promising strategy to contrast neurodegeneration and cognitive symptoms (11). Consistently, endogenous lipids belonging to the N-acyl-ethanolamine (NAE) fatty acid amide family, such as anandamide (AEA), oleoylethanolamide (OEA), and palmitoylethanolamide (PEA), have shown the ability to mitigate pathogenetic mechanisms involved in the neurodegeneration process (12). PEA was initially discovered in egg yolk, soybean, and peanut oil and, later, in mammalian tissues. While exerting cannabimimetic action, it does not bind to cannabinoid receptors (13). Instead, it activates the peroxisome proliferator-activated receptor-α (PPAR-α) as well as its associated independent pathways, including ion channels involved in neuronal firing and the Transient Receptor Potential Vanilloid 1 (TRPV1) receptor (14). Such peculiar activity is believed to explain PEA potential anti-inflammatory, analgesic, and anti-epileptic effects (15–18). Interestingly, several PEA-containing products are licensed as nutraceuticals or food supplements for human use in different countries, at a recommended dose of 600–1,200 mg/day (19).

Accumulating evidence suggests that PEA may play a role in counteracting neurodegenerative processes (20), by modulating neuroinflammation pathways such as astrocyte and microglia proliferation and neuronal loss (21). Thus, PEA may be a promising therapeutic option to contrast inflammatory and oxidative stress, with potential effects in the treatment of neurodegeneration processes. Within this systematic review, we tried and better clarify the role of PEA in the context of NCDs and cognitive decline by gathering and discussing all available data from clinical and preclinical research, including both interventional and observational studies.

## Methods

### Inclusion and exclusion criteria

In order to bring together previous evidence on the topic, inclusion criteria were used as follows: (1) human or animal studies, (2) studies investigating palmitoylethanolamide (PEA) effects over primary (e.g., Alzheimer's disease), secondary (e.g., Parkinson's disease) or acquired (e.g., Traumatic Brain Injury) cognitive decline associated with neurocognitive disorders (NCDs), (3) studies investigating PEA effects over cognitive decline associated to conditions (e.g., neuropathic pain, obesity) other than NCDs, (4) studies investigating PEA modulatory effects over the biological underpinnings (e.g., neuroinflammation, gliosis, neuronal death) of cognitive decline in the context of NCDs, (5) studies investigating PEA and PEA signaling-related molecular markers (e.g., brain and/or other tissue quantitative alterations) of cognitive decline in the context of NCDs. Exclusion criteria were (1) studies investigating neither PEA as the intervention of interest (e.g., studies evaluating only exogenous cannabinoid agonists or antagonists) nor PEA or PEA signaling-related molecular markers, (2) studies where PEA bio-cognitive correlates were not investigated with reference to NCDs nor other conditions associated with cognitive decline, and (3) studies where PEA bio-cognitive correlates were not directly reported on.

### Search strategy and data extraction

A literature search was performed using electronic databases (Pubmed, Web of Science, and Scopus) for any published original study written in English, using a combination of terms concerning PEA (“palmitoylethanolamide,” “palmitylethanolamide,” “N-(2-hydroxyethyl)hexadecanamide,” “N-(2-hydroxyethyl)palmitate” and “N-palmitoylethanolamine”) and NCDs (“dementia,” “memory,” “cognit^*^”, “executive function,” “neurocognitive disorder,” “attenti^*^”, “learning,” “language,” “sensory-motor” and “neurodegenerati^*^”) on 25 May 2022. Broad-meaning terms were used to make the study search as inclusive as possible. Reference lists of eligible studies were screened to identify additional eligible research. Publication data screening and extraction were performed following a conventional double-screening process independently conducted by two reviewers (R.B. and C.C.).

### Risk of bias

Due to the methodological heterogeneity of the studies (Table 1) included in this review, risk of bias and study quality assessments were conducted with a reasonably inclusive and flexible approach, in line with previous research in the field (15, 53). To this extent, interventional and observational studies in humans were evaluated through an adapted set of criteria suggested by the Agency for Healthcare Research and Quality (AHRQ) guidance (54), and risk of systematic bias across human studies was further ruled out by screening all papers for potential confounding variables, such as patients' age and educational level (Table 2). Moreover, factors possibly accounting for similarities and differences between animal studies were assessed, extracting information about study characteristics, including animal model (e.g., mouse or rat), developmental stage (e.g., postnatal, adult, primary cultures of astrocytes or neurons), gender, PEA measure (e.g., PEA dosage and administration route, PEA assessment in tissues) and adequate PEA evaluation (e.g., time of exposure, single or multiple tissue assessments) (Table 2).

**Table 1A:** Summary of clinical studies investigating palmitoylethanolamide and its correlations to neurocognitive disorders (NCDs).

**References (Country)**	**Aim of study**	**Type of study**	**Population**	***N***	**Outcome measure (test name or description)**	**Summary results**	**Additional information of interest**
Paterniti et al. (22) (Italy)	To assess PEA effects on Aβ exposed human neuronal cells	*In vitro* exposure in humans	1. CTRL; 2. Aβ; 3. Aβ+PEA(0.27 + 0.027 μM); 4. Aβ+PEA(2.7 + 0.27 μM); 5. Aβ+PEA(27 + 2.7 μM)	X	1. Effects on neuronal viability (Vital staining); 2. Effects on brain function (Measurement fo nitrite concentration, Western blot, Alcaline Comet-assay)	PEA administration restores IκBα level and NFκB nuclear translocation in *in vitro* neuronal cells after Aβ exposure	/
Altamura et al. (23) (Italy)	To assess eCBs/AEs levels modulation in AD patients	Quantitative assessment in humans	1. AD; 2. CTRL	71	1. eCBs/AEs levels (Blood sample); 2. Carotid atherosclerosis markers (continuous wave Doppler, Color flow B-mode Doppler ultrasound); 3. Memory and cognition (MMSE, Rey Auditory Verbal Learning, oral denomination test, Raven's Colored Progressive Matrices); 4. Neuroradiological evaluation (MRI)	1. PEA blood levels are not significantly higher in AD patients compared to controls; 2. Higher PEA blood levels are related to lower constructional praxia test score	1.2-AG blood levels are higher in AD patients compared to controls; 2. 2-AG blood levels are positively related to memory, attention and WMH volume in AD patients; 3. 2-AG blood levels are higher in AD patients with chronic heart ischemic disease; 4. AEA and OEA blood levels are not significantly higher in AD patients compared to controls
Caltagirone et al. (24) (Italy)	To assess PEA effects on memory and cognitive function in stroke patients	*In vivo* exposure in humans	Ischemic stroke patients	250	1. Neurological condition (CNS); 2. Memory and cognition (MMSE)	1. PEA administration ameliorates neurological status after 30-day treatment in ischemic stroke patients; 2. PEA administration ameliorates cognitive impairment after 30-day treatment in ischemic stroke patients; 3. PEA is well tolerated with no side events all over the time of the study in stroke patients	PEA administration improves spasticity, pain and independence in daily living after 30-day treatment in ischemic stroke patients
Cipriano et al. (25) (Italy)	To assess PEA anti-inflammatory and anti-angiogenic effects on Aβ-exposed HUVEC cells	*In vitro* exposure in humans	1. CTRL; 2. Aβ; 3. Aβ+PEA10^∧^−6; 4. Aβ+PEA10^∧^−7; 5. Aβ+PEA10^∧^−8; 6. Aβ+PEA10^∧^6+GW6471(2.5); 7. Aβ+PEA10^∧^−6+GW6471(5); 8. Aβ+PEA10^∧^−6+GW6471(10)	X	1. Cell viability (Cell Vitality Assay); 2. Effect on pro-angiogenic factors production and release (Western blot, ELISA); 3. Effect on endothelial cell proliferation (Immunofluorescence, ELISA BrdU assay, ATP Bioluminescence assay)	1. PEA administration reduces HUVEC cell proliferation; 2. PEA effect is counteracted by GW6471 administration	/
Brotini et al. (26) (Italy)	To assess PEA effects on non-motor symptoms in PD patients	*In vivo* exposure in humans	PD patients	30	Non-motor Aspects of Experiences of Daily Living (MDS-UPDRS)	1. PEA add-on to levodopa ameliorates several nM-EDL symptoms in PD patients; 2. PEA is well tolerated with no side events all over the time of the study in PD patients	PEA add-on to levodopa ameliorates almost all M-EDL symptoms in PD patients
Assogna et al. (27) (Italy)	To assess PEA effects on memory, cognitive function and frontal lobe activity in FTD patients	*In vivo* exposure in humans	FTD patients	17	1. Behavior, memory and cognition (NPI, MMSE, FAB, SAND); 2. Independency (ADL/IADL); 3. Neurological condition (FTLD-CDR); 4. Corticospinal evaluation (TMS); 5. TMS-EEG cortical evaluation	1. PEA improves frontal lobe functions in FTD patients; 2. PEA reduces behavioral disturbances in FTD patients; 3. PEA restores LICI at ISI 100 in FTD patients; 4. PEA leads to an increase in TMS-evoked frontal lobe activity and high-frequency oscillations in the beta/gamma range; 5. PEA is well tolerated with no side events all over the time of the study in FTD patients	/
Campolo et al. (28) (Italy)	To assess PEA effects on memory and cognitive function in TBI patients	*In vivo* exposure in humans	1. PEA+std; 2. std	30	1. TBI severity (GCS, Marshal Score); 2. Memory and cognition (MMSE, BNCE); 3. Depressive symptoms (BDI); 4. Independency (Barthel Index)	1. PEA add-on improves memory and cognitive function compared to standard monotherapy in TBI patients; 2. PEA is well tolerated with no side events all over the time of the study in TBI patients	1. PEA add-on ameliorates independence and mobility in quotidian living activities compared to baseline in TBI patients; 2. PEA add-on does not improve significantly depressive symptoms compared to standard monotherapy in TBI patients

PEA, palmitoylethanolamide; Aβ, β-amyloid precursor protein; CTRL, control; μM, micromolar; IκBα, nuclear factor of kappa light polypeptide gene enhancer in B-cells inhibitor, alpha; NFκB, nuclear factor kappa-light-chain-enhancer of activated B cells; eCBs, endocannabinoids; AEs, acylethanolamines; AD, Alzheimer's disease; MMSE, Mini Mental State Examination; MRI, Magnetic resonance imaging; 2-AG, 2-arachidonoylglycerol; WMH, white matter hyperintensity; AEA, anandamide; OEA, oleoylethanolamide; CNS, Canadian Neurological Scale; HUVEC, Human umbilical vein endothelial cells; GW6471, PPARα antagonist; ELISA, Enzyme-linked immunosorbent assay; BrdU, Bromodeoxyuridine; ATP, Adenosine triphosphate; PD, Parkinson's disease; MDS-UPDRS, Movement Disorder Society/Unified Parkinson's Disease Rating Scale; nM-EDL, Non-motor Aspects of Experiences of Daily Living; M-EDL, Motor Aspects of Experiences of Daily Living; FTD, Frontotemporal dementia; NPI, Neuropsychiatric Inventory; FAB, Frontal Assessment Battery; SAND, Screening for Aphasia in Neurodegeneration; ADL, Activities of Daily Living; IADL, Instrumental Activities of Daily Living; FTLD-CDR, Frontotemporal Lobar Degeneration-modified Clinical Dementia Rating scale; TMS, transcranial magnetic stimulation; TMS-EEG, TMS combined with electroencephalography; LICI, long-interval intracortical inhibition; ISI, inter-stimulus-interval; TBI, traumatic brain injury; std, standard; GCS, Glasgow coma scale; BNCE, brief neuropsychological cognitive examination; BDI, Beck's inventory depression scale.

**Table 1B:** Summary of preclinical studies investigating palmitoylethanolamide and its correlations to neurocognitive disorders (NCDs).

**References (Country)**	**Aim of study**	**PEA type of study**	**Population**	**N**	**Outcome measure (test name or description)**	**Summary results**
Scuderi et al. (29) (Italy)	To assess PEA effects on Aβ-exposed rat astrocytes	*In vitro* exposure in animals	1. CTRL; 2. Aβ; 3. Aβ+PEA; 4. Aβ+PEA+MK; 4. Aβ+PEA+GW9662	X	1. Astrocytes activation (Western blot, Immunofluorescence, RT-PCR, densitometric analysis, ELISA); 2. Neuroinflammation (Western blot, Immunofluorescence, spectrophotometric assay based on the Griess reaction, ELISA); 3. Anti-inflammatory effects (Western blot, EMSA analysis); 4. Effects on eCB system (Western blot analysis and densitometric analysis)	1. PEA application reduces Aβ-induced neuroinflammation and astrocytes' activation; 2. PEA effects on atrocytes are counteracted by MK886 administration; 3. PEA application increases PPAR-α, CB1 and CB2 expression after Aβ exposure in astrocytes
Benito et al. (30) (Italy)	To assess PEA effects on Aβ-exposed FAAH-KO mice astrocytes	*In vitro* exposure in animals	1. FAAH-WT group: (a) CTRL-WT; (b) Aβ-WT; (c) PEA; (d) Aβ+PEA; (e) Aβ+PEA+AEA+OEA; (f) OEA; (g) AEA; (h) Aβ+OEA; (i) Aβ+AEA; (j) URB; (k) Aβ+URB; (l) URB+SR1; (m) Aβ+URB+SR1; (n) URB+SR2; (o) Aβ+URB+SR2; 2. FAAH-KO group: (a) CTRL-KO; (b) Aβ-KO; (c) SR1; (d) Aβ+SR1; (e) SR2; (f) Aβ+SR2; (g) WY; (h) Aβ+WY; (i) TG; (j) Aβ+TG; (k) CPZ; (l) Aβ+CPZ	X	1. Anti-inflammatory effects (ELISA, Western blot, qRT-PCR); 2. Cell death (LDH dosage)	PEA alone or combined with other eCBs/AEs decreases Aβ-induced inflammatory effects in astrocytes
D'Agostino et al. (31) (Italy)	To assess PEA effects on cognitive function and neuroprotection in AD mice	*In vivo* exposure in animals	1. First set of mice (WT, PPARα-/-): (a) ScAb+VHI; (b) Ab+VHI; (c) Ab+PEA3; (d) Ab+PEA10; (e) Ab+PEA30; (f) Ab+GW7647; 2. Second, 3. Third sets of mice (WT): (a) ScAb+VHI; (b) Ab+VHI; (c) Ab+PEA30	8–10 per group	1. Memory and cognition (YMT, MWM, WMT, NORT, Rotarod test); 2. Effects on brain function (Western blot, Lipid Peroxidation Measures)	PEA administration restores learning and memory impairment and exerts a neuroprotective action at high dose in AD mice
Scuderi et al. (32) (Italy)	To assess PEA effects on Aβ-exposed rat neurons and astrocytes	1. *Ex vivo* exposure in animals; 2. *In vitro* exposure in animals	1. CTRL; 2. Aβ; 3. Aβ+PEA; 4. Aβ+PEA+MK; 5. Aβ+PEA+GW6471	X	Astrocyte proliferation and neuronal loss (Nissl staining, Immunofluorescence)	PEA application blunts Aβ-induced astrocyte activation and exerts a protective effect on neurons in rats
Scuderi and Steardo (33) (Italy)	To assess PEA effects on Aβ-exposed hippocampal tissue and neurons in rats	1. *Ex vivo* exposure in animals; 2. *In vitro* exposure in animals	1. Hippocampal slice cultures, 2. Cultures of primary neurons: (a) CTRL; (b) Aβ; (c) Aβ+PEA; (d) Aβ+PEA+GW6471	X	1. Hippocampal tissue functioning, 2. Neuroinflammation (Nissl staining, Immunofluorescence, Western blot, ELISA); 3. Neuronal viability (Neutral red assay)	PEA application blunts Aβ-induced astrocyte activation and exerts a protective effect on neurons in rats
Paterniti et al. (22) (Italy)	To assess PEA effects on Aβ-exposed mouse brain tissue	*Ex vivo* exposure in animals	1. CTRL; 2. Aβ; 3. Aβ+PEA(0.27 + 0.027 μM); 4. Aβ+PEA(2.7 + 0.27 μM); 5. Aβ+PEA(27 + 2.7 μM)	X	1. Effects on neuronal viability (Vital staining); 2. Effects on brain function (Measurement fo nitrite concentration, Western blot, Comet analysis)	1. PEA administration increases neuronal viability in Aβ exposed mouse hippocampus; 2. PEA administration restores BDNF and GDNF levels in Aβ exposed mouse hippocampus; 3. PEA administration reduces GFAP activation in Aβ exposed mouse hippocampus; 4. PEA administration decreases nitrite production in Aβ exposed mouse hippocampus; 5. PEA administration rescues programmed cellular death in Aβ exposed mouse hippocampus; 6. PEA administration reduces DNA damage in Aβ exposed mouse hippocampus
Scuderi et al. (34) (Italy)	To assess PEA anti-inflammatory and neuro-protective effects in Aβ-exposed rats	*In vivo* exposure in animals	1. VHI; 2. VHI+Aβ; 3. PEA+VHI; 4. PEA+Aβ; 5. PEA+GW6471+VHI; 6. PEA+GW6471+Aβ	9–12 per group	1. Glia activation, 2. Neuroinflammation (RT-PCR, Immunofluorescence, Western blot, ELISA); 3. Effect on the amyloidogenic and Wnt pathway (Western blot); 4. Neuronal viability (Immunofluorescence); 5. Memory and cognition (MWM)	1. PEA administration counteracts Aβ-induced reactive gliosis and amyloidogenesis in rats; 2. PEA administration improves neuronal integrity after Aβ-exposure in rats; 3. PEA administration prevents Aβ-induced memory impairment in rats; 4. PEA exerts neuroprotective and anti-inflammatory effects through PPAR-α activation
Cipriano et al. (25) (Italy)	To assess PEA anti-inflammatory and anti-angiogenic effects on Aβ-exposed rat glioma cells	*In vitro* exposure in animals	1. CTRL; 2. Aβ; 3. Aβ+PEA10^∧^−6; 4. Aβ+PEA10^∧^−7; 5. Aβ+PEA10^∧^−8; 6. Aβ+PEA10^∧^−6+GW6471(2.5); 7. Aβ+PEA10^∧^−6+GW6471(5); 8. Aβ+PEA10^∧^−6+GW6471(10)	X	1. Glia activation (Cell Vitality assay, Griess reaction, Western blot); 2. Effect on pro-angiogenic factors production and release (Western blot, ELISA)	PEA concentration-dependently reduces the expression of 1. pro-inflammatory and 2. pro-angiogenic markers in Aβ treated cells.
Tomasini et al. (35) (Italy)	To assess PEA effects on Aβ exposed AD mouse neurons and astrocytes	*In vitro* exposure in animals	1. Primary cerebral cortex neurons (3xTg-AD, Non-Tg): (a) CTRL; (b) PEA; (c) Aβ; (d) PEA+Aβ; 2. Primary cerebral cortex astrocytes (3xTg-AD, Non-Tg): (a) CTRL; (b) PEA; (c) Aβ; (d) PEA+Aβ	X	1. Cell viability (Neutral red assay); 2. Endogenous extracellular glutamate levels (High-performance liquid chromatography/fluorimetric detection system); 3. Cell morphology (Immunocytochemistry)	PEA administration exerts protective properties in Non-Tg but not in 3xTg-AD Aβ-exposed mouse neuronal cultured cells
Caltagirone et al. (24) (Italy)	To assess PEA neuro-protective and behavioral effects in MCAo rats	*In vivo* exposure in animals	1. MCAo+VHI; 2. MCAo+PEA; 3. sham+VHI; 4. sham+PEA	1. First set of experiment: 20 per group; 2. Second set of experiment: 10 per group	1. First set of experiment: (a) Motor behavior (Mean rotation number/h, Neurological scoring); (b) Brain tissue damage (Histological evaluation); 2. Second set of experiment: (a) Astrocyte activation (Immunohistochemistry, Western blot); (b) BDNF, GDNF expression (Western blot); (c) Mast cells infiltration, (d) Enzymatic expression (Immunohistochemistry); (e) Programmed cell death (Western blot)	PEA administration improves neurobehavioural function, reduces neuroinflammation and counteracts histological damage in ischemic rats
Siracusa et al. (36) (Italy)	To assess PEA anti-inflammatory and neuroprotective effects in VaD mice	1. *In vivo* exposure in animals; 2. Quantitative brain assessment	1. Healthy rats: only used to test PEA pharmacokinetics; 2. Mice: (a) sham+VHI; (b) sham+PEA; (c) VaD+VHI; (d) VaD+PEA	40 (10 per group)	1. PEA brain levels (LC-APCI-MS); 2. Memory and cognition (NORT); 3. Social behavior (Social Interaction test); 4. Locomotor activity (OFT); 5. Effects on brain function (Immunohistochemistry, Immunofluorescence, Western blot)	1. PEA oral administration results in low-medium PEA brain concentrations shortly after in healthy rats; 2. PEA administration rescues injured hippocampal CA1 and CA3 neurons in VaD mice; 3. PEA administration rescues impaired memory, social behavior and locomotor activity in VaD mice; 4. PEA administration exerts anti-inflammatory and neuroprotective effects in VaD mice
Beggiato et al. (37) (Italy)	To assess PEA effects on Aβ exposed mouse neurons and astrocytes	*In vitro* exposure in animals	1. CTRL; 2. Aβ; 3. Aβ+PEA; 4. PEA	X	1. Cell viability (Neutral red assay); 2. Cell morphology (Immunofluorescence); 3. Programmed cell death (% of neurons' apoptotic nuclei, DNA staining)	PEA administration improves neuronal survival and morphology, by blunting Aβ-induced mouse astrocyte activation
Bronzuoli et al. (38) (Italy)	To assess PEA anti-inflammatory and neuro-protective effects in AD mice	1. *In vivo* exposure in animals; 2. *In vitro* exposure in animals	1. *In vivo* (*n* = 18 3xTg-AD, *n* = 18 Non-Tg): (a) CTRL; (b) PEA(0.01); (c) PEA(0.1); (d) PEA(1); 2. *In vitro* (*n* = 36 3xTg-AD, *n* = 12 Non-Tg): (a) CTRL; (b) PEA	84	1. Primary astrocytes activation (Immunofluorescence, Western blot); 2. Astrocytes and neuronal viability (Neutral red assay); 3. Reactive astrogliosis, 4. Neuronal support and survival (RNA isolation, RT-PCR, Western blot, Immunofluorescence)	PEA *in vitro* application and *in vivo* administration supports neuronal viability and reduces gliosis in AD mice
Crupi et al. (39) (Italy)	To assess PEA anti-inflammatory and neuroprotective effects in PD mice	*In vivo* exposure in animals	1. sham+VHI; 2. sham+PEA; 3. MPTP+VHI; 4. MPTP+PEA	40 (10 per group)	Effects on brain function (Immunofluorescence)	PEA administration prevents the decrease in adult hippocampal cell proliferation and β3-tubulin aggregation in PD mice
Scuderi et al. (20) (Italy)	1. To assess chronic PEA effects on cognitive function in AD mice; 2. To assess chronic PEA effects on brain function in AD mice	*In vivo* exposure in animals	1. First set of mice (3 months): (a) PEA (3 × Tg-AD/Non-Tg); (b) placebo (3 × Tg-AD/Non-Tg); 2. Second set of mice (9 months): (a) PEA (3 × Tg-AD/Non-Tg); (b) placebo (3 × Tg-AD/Non-Tg)	1. First set of mice (3 months): 9–11 per group; 2. Second set of mice (9 months): 7–9 per group	1. Memory and cognition (NORT, IA, MWM); 2. Depressive-/Anhedonia-like behavior (TST, FST, SPT); 3. Effects on brain function (RT-PCR, Western blot, Immunohistochemistry, Cytokine array, HPLC, MRI/MRS)	1. PEA administration rescues early learning and memory deficits in 6-mo AD mice; 2. PEA administration improves short-term memory in 12-mo AD mice, with no significant effects on long-term memory; 3. PEA administration reverses the depressive-like phenotype in 6-mo AD mice, with no significant effects in 12-mo AD mice; 4. PEA administration attenuates the anhedonia-like phenotype in 6- and 12-mo AD mice; 5. PEA administration reduces hippocampal Aβ expression in 12-mo AD mice, with no significant effects in 6-mo AD mice; 6. PEA administration reduces abnormal hippocampal tau phosphorylation in 6- and 12-mo AD mice; 7. PEA administration promotes MAP2 expression in the CA1 subregion of hippocampus of AD mice; 8. PEA administration stabilizes astrocyte function and restrains neuroinflammation in AD mice; 9. PEA administration increases Glx levels as a response to disrupted glutamatergic functionin 6-mo AD mice
Boccella et al. (40) (Italy)	To assess PEA effects on cognitive function and their mGluR-mediated modulation in SNI mice	*In vivo* exposure in animals	1. sham: (a) VHI; (b) PEA; (c) MPEP; (d) MPEP+PEA; (e) MDCPG; (f) MDCPG+PEA; 2. SNI: (a) VHI; (b) PEA; (c) MPEP; (d) MPEP+PEA; (e) MDCPG; (f) MDCPG+PEA	96	Memory and cognition (NORT)	1. PEA administration rescues discriminative memory in SNI mice; 2. PEA beneficial effects on discriminative memory are prevented by the mGluR5 blockade, but not the mGluR8 blockade in SNI mice
Boccella et al. (41) (Italy)	To assess PEA effects on cognitive function in SNI mice	1. *In vivo* exposure in animals; 2. Quantitative brain assessment	1. sham+VHI; 2. sham+PEA; 3. SNI+VHI; 4. SNI+PEA	10 per group	1. Memory and cognition (NORT, MWM); 2. Effects eCBs/AEs system (LC-APCI-MS)	1. PEA administration rescues discriminative and spatial memory deficits in SNI mice, by restoring LTP and synaptic maladaptative changes in the LEC-DG pathway; 2. PEA administration affects 2-AG, but not PEA nor AEA LEC levels in sham and SNI mice
Impellizzeri et al. (42) (Italy)	To assess PEA anti-inflammatory and neuroprotective effects in VaD mice	1. *In vivo* exposure in animals; 2. Quantitative brain assessment	1. sham+VHI; 2. sham+PEA; 3. VaD+VHI; 4. VaD+PEA	40 (10 per group)	1. Memory and cognition (NORT, MWM); 2. Effects on brain function and 3. eCBs/AEs system (Light microscospy, Immunohistochemistry, Immunofluorescence, TUNEL staining, LP-APCI-MS, Western blot)	1. Endogenous PEA levels decrease after VaD induction; 2. PEA administration increases PEA endogenous levels in VaD mice; 3. PEA administration rescues injured hippocampal CA1 and CA3 neurons in VaD mice; 4. PEA administration exerts anti-inflammatory and neuroprotective effects in VaD mice; 5. PEA administration rescues learning and memory deficits in VaD mice
Piscitelli et al. (43) (Italy)	To assess PEA and other eCBs/AEs brain and plasma levels in AD-like Tg mice	Quantitative tissue assessment	1. WT; 2. Tg	10	1. Brain tissue eCBs/AEs levels (LP-APCI-MS); 2. Plasma levels	1. PEA and other eCBs/AEs levels are not altered in AD-like Tg mouse model compared to WT mice; 2. PEA and other eCBs/AEs levels show no overt alterations from presymptomatic, mild symptomatic to symptomatic disease stages in AD-like Tg mouse model
Zimmermann et al. (44) (Germany)	To assess PEA/AEA signaling alterations and related effects on cognitive function in AAV-Glu-FAAH mice	Quantitative brain assessment	1. AAV-Glu-FAAH; 2. AAV-Glu-empty; 3. AAV-WT	3–16 per group	1. Memory and cognition (spatial object recognition test); 2. PEA and other AEs brain levels (LC-MS/MS)	Impaired PEA signaling in hippocampal glutamatergic neurons alters synaptic plasticity, learning, and emotional responses
Beggiato et al. (45) (Italy)	To assess PEA neuroprotective effects in AD mice	*In vitro* exposure in animals	Mature cerebral cortex astrocytes: 1. Non-Tg: (a) CTRL; (b) Aβ; (c) Aβ+PEA; 2. 3xTg-AD: (a) CTRL; (b) Aβ; (c) Aβ+PEA	4–5 animals per condition	1. Effects on neuronal viability (Neutral red assay); 2. Effects on neuronal morphology (Immunohistochemistry); 3. Effects on apoptotic neuronal death (Immunofluorescence)	PEA application prevents Aβ-induced astrogliosis, thus improving neuronal survival in AD mice
Beggiato et al. (46) (Italy)	1. To assess PEA effects on cognitive function in AD mice; 2. To assess PEA anti-inflammatory and neuroprotective effects in AD mice; 3. To assess PEA effects on glutamate levels in AD mice	1. *In vivo* exposure in animals; 2. Quantitative tissue assessment	1.3 × Tg-AD+VHI; 2. 3 × Tg-AD+PEA; 3. Non-Tg+VHI; 4. Non-Tg+PEA	4–11 per group	1. Memory and cognition (NORT); 2. Effects on neuroinflammation, 3. Effects on neuroprotective factors expression (Immunofluorescence); 4. Hippocampal glutamate levels (HPLC coupled to fluorescence detection)	1. PEA administration improves learning and memory in 5-mo AD mice; 2. PEA administration partially restrains neuroinflammation in 5-mo AD mice; 3. PEA administration reduces oxidative stress in 5-mo AD mice; 4. PEA administration does not affect Synaptophysin hippocampal levels in 5-mo AD mice; 5. PEA administration partially rescues increased glutamate levels in the hippocampus of 5-mo AD mice
Facchinetti et al. (47) (Italy)	To assess PEA anti-inflammatory and neuroprotective effects in prodromal AD rats	*In vivo* exposure in animals	1. VHI; 2. VHI(Aβ); 3. PEA(VHI); 4. PEA(Aβ)	4–5 per group	Effects on brain function (Immunofluorescence, qRT-PCR)	1. Early PEA administration prevents Aβ-induced astrogliosis and microgliosis in AD rats; 2. Early PEA administration prevents the increased gene expression of pro-inflammatory cytokines and enzymes in AD rats; 3. Early PEA administration improves hippocampal neuronal survival in AD rats
Lama et al. (48) (Italy)	To assess PEA effects on cognitive function in HFD mice	*In vivo* exposure in animals	1. STD; 2. HFD; 3. HFD+PEA	≥ 15 per group	Memory and cognition (NORT)	PEA administration restores recognition memory in HFD mice
Boccella et al. (49) (Italy)	To assess PEA effects on cognitive function in SNI mice	*In vivo* exposure in animals	1. sham+VHI; 2. sham+PEA; 3. SNI+VHI; 4. SNI+PEA	120	Memory and cognition (MWM, Y-maze)	PEA administration rescues spatial memory and working-memory in SNI mice
Campolo et al. (28) (Italy)	1. To assess PEA anti-inflammatory and neuroprotective effects in TBI mice; 2. To assess PEA effects on cognitive function in TBI mice	*In vivo* exposure in animals	1. sham; 2. sham+PEA; 3. TBI; 4. TBI+PEA	40 (10 per group)	1. Memory and cognition (MWM); 2. Effects on brain function (Histological analysis, Immunohistochemistry, Immunofluorescence, FluoroJade, Western blot)	1. PEA administration rescues learning and memory deficits in TBI mice; 2. PEA administration modulates neurogenesis processes in TBI mice; 3. PEA administration accelerates NSCs proliferation in TBI mice
D'Antongiovanni et al. (50) (Italy)	To assess PEA effects on enteric inflammation and bowel motor dysfunctions in AD mice	1. *In vivo* exposure in animals; 2. *In vitro* exposure in animals	1. *In vivo*/*In vitro* exposure: (a) SAMR1; (b) SAMP8; (c) SAMP8+PEA; 2. *In vitro* exposure: (a) CTRL; (b) LPS+Aβ; (c) LPS+Aβ+PEA	X	1. Effects on colonic contractile activity (ES, chemical stimulation); 2. Effects on misfolded proteins (ELISA assay); 3. Effects on enzymatic activity (Enzymatic assay); 4. Effects on colonic inflammation (ELISA, Western blot)	1. PEA administration/application prevents the enteric glial hyperactivation in AD mice; 2. PEA administration/application reduces misfolded protein accumulation and counteracts colonic inflammatory condition in AD mice; 3. PEA administration/application relieves intestinal motor dysfunctions in AD mice; 4. PEA administration/application improves the intestinal epithelial barrier integrity in AD mice
Gaspar et al. (51) (Ireland)	1. To assess PEA effects on inflammatory pain-related cognitive impairment in CFA-treated rats; 2. To assess PEA and other AEs brain levels in CFA-treated rats	1. *In vivo* exposure in animals; 2. Quantitative brain assessment	1. noCFA, 2. CFA: (a) VHI; (b) GW6471; (c) GSK; (d) GW9662; (e) PEA	80	1. Memory and cognition (NORT); 2. PEA and other AEs brain levels (LC-MS/MS)	1. PPARα antagonist impairs spatial memory in CFA-treated rats; 2. PEA levels are not modified in the Dorsal Hippocampus nor in the Entorhinal Cortex of CFA-Injected rats
Gatta et al. (52) (Italy)	To assess PEA anti-inflammatory and neuroprotective effects in AD-like mouse microglial cells	1. *In vitro* exposure in animals; 2. *Ex vivo* exposure in animals	1. BV2 microglial cell model: (a) CTRL; (b) LPS; (c) LPS+PEA; (d) Aβ; (e) Aβ+PEA; 2. Mature cerebral cortex microglial cells: (a) CTRL; (b) LPS; (c) LPS+PEA	4–6 animals per condition	Effects on brain function (Western blot, semi-quantitative qRT-PCR)	PEA reduces LPS- or Aβ-induced neuroinflammation and TG2 overexpression in mouse microglial cells

PEA, palmitoylethanolamide; Aβ, β-amyloid precursor protein; CTRL, control; MK, MK886 (PPARα antagonist); GW9662, PPARγ antagonist; RT-PCR, Reverse transcriptase-PCR analysis; ELISA, Enzyme-linked immunosorbent assay; EMSA, Electrophoretic mobility shift assay; eCB, endocannabinoid; FAAH, Fatty acid amide hydrolase; KO, Knock-out; WT, Wild-type; AEA, anandamide; OEA, oleoylethanolamide; URB, URB597; SR1, SR141716A; SR2, SR144528; WY, WY14643; TG, troglitazone; CPZ, capsazepine; qRT-PCR, RT quantitative-PCR; LDH, Lactate dehydrogenase; AE, acylethanolamine; AD, Alzheimer's disease; PPARα, Peroxisome proliferator-activated receptor alpha; ScAb, Scrambled Ab25-35 peptide; VHI, vehicle; Ab, Ab25-35 peptide; PEA(3, 10, 30), PEA (3, 10, 30 mg/Kg); GW7647, PPARα agonist; YMT, Y-Maze test; MWM, Morris Water Maze test; WMT, Working-memory test; NORT, Novel Object Recognition test; GW6471, PPARα antagonist; μM, micromolar; BDNF, Brain-Derived Neurotrophic Factor; GDNF, Glial cell line-derived neurotrophic factor; GFAP, Glial fibrillary acidic protein; DNA, Deoxyribonucleic acid; 3xTg-AD, triple-transgenic mouse model of AD; non-Tg, non-transgenic mouse model; MCAo, middle cerebral artery occlusion; VaD, vascular dementia; LC-APCI-MS, Liquid chromatography-atmospheric pressure chemical ionization-mass spectrometry; OFT, Open-field test; RNA, Ribonucleic acid; PD, Parkinson's disease; MPTP, 1-methyl-4-phenyl-1,2,3,6-tetrahydropyridine; IA, Inhibitory passive avoidance; TST, Tail suspension test; FST, Forced swim test; SPT, Sucrose preference test; HPLC, High-performance liquid chromatography; MRI, Magnetic resonance imaging; MRS, Magnetic resonance spectroscopy; -mo, month-old; MAP2, Microtubule-associated protein 2; Glx, Glutamine/glutamate; mGluR, Metabotropic glutamate receptor; SNI, spare nerve injury; MPEP, 2-Methyl-6-(phenylethynyl) pyridine; MDCPG, (RS)-4-(1-amino-1-carboxyethyl)phthalic acid; LTP, long-term potentiation; LEC, lateral entorhinal cortex; DG, dentate gyrus; 2-AG, 2-arachidonoylglycerol; eCBs, endocannabinoids; AEs, acylethanolamines; Tg, transgenic; AAV, adeno-associated virus; Glu, glutamatergic neurons; AAV-Glu-FAAH, Animals overexpressing FAAH in glutamatergic neurons; LC-MS/MS, liquid chromatography–mass spectrometry; HFD, high-fat diet; TBI, traumatic brain injury; NSCs, neuronal stem cells; SAMR1, Senescence-Accelerated Mouse-Resistant 1; SAMP8, Senescence Accelerated Mouse-prone 8; LPS, lipopolysaccharide; ES, electrical stimulation; CFA, Complete Freund's Adjuvant; GSK, GSK0660 (PPARβ/δ antagonist); TG2, Tissue type 2 transglutaminase.

**Table 2A:** Methodological quality of clinical studies investigating palmitoylethanolamide and its correlations to neurocognitive disorders (NCDs).

**References (Country)**	**Study design**	**Defined study population**	**Age (years)**	**Gender**	**PEA measure**	**Adequate PEA evaluation**	**Control**	**Comparability of subjects**	**Other comorbidity**	**Excluded/adjusted for confounding factors**	**Statistical analyses**	**Funding or sponsorship**
Paterniti et al. (22) (Italy)	√ Analytic, observational, interventional	√ SH-SY5Y neuroblastoma differentiated neuron-like cells	X	X	√ co-ultra PEALut (um-PEA 0.27 μM + luteolin 0.027 μM or um-PEA 2.7 μM + luteolin 0.27 μM or um-PEA 27 μM + luteolin 2.7 μM) *in vitro* addition	√ Single application (added to medium 2 h before injury)	1. CTRL; 2. Aβ	√ Experimental condition	√ No comorbidity	√ No exclusion criteria; no confounders	√ ANOVA, Bonferroni's test	X
Altamura et al. (23) (Italy)	√ Analytic, observational	√ AD patients dementia therapy naive	√ 1. AD: 77.3 ± 6.4; 2. CTRL: 75 ± 3.6	√ Male and female	√ Blood levels	√ Single assessment	√ CTRL	√ Age; gender	√ Obesity; Smoking habit; Diabetes; Hypertension; Hyperlipidemia; Chronic heart ischemic disease; ApoEε4	√ Excluded if: (a) history or signs of previous stroke or other neurological diseases; (b) chronic or recurrent acute pain; (c) use of cannabinoids for recreational or medical purposes; (d) acute infectious disease; (e) alcohol abuse; (f) history of systemic inflammatory and neoplastic diseases; Adjusted for: (a) age; (b) educational level (Memory and cognition assessments)	√ Student's *t*-test, Mann-Whitney U test, χ2 test, Kolmogorov–Smirnov test, ANOVA, Spearman's rho	X
Caltagirone et al. (50) (Italy)	√ Analytic, observational, interventional	√ First ischemic stroke stabilized patients undergoing rehabilitative therapy	√ 71.4 ± 12.4	√ Male and female	√ co-ultra PEALut (um-PEA 700 mg + luteolin 70 mg) sublingual administration	√ Bid administration (60 days)	X	√ Clinical condition; age range	√ No comorbidity	√ Excluded if: (a) previously hospitalized stroke patients; (b) hemorrhagic stroke patients; (c) bilateral stroke patients; (d) no first ischemic stroke patients; (e) ≥ 18 months before ischemic event; (f) inadequate information about ischemic event; Adjusted for: (a) age; (b) educational level (MMSE)	√ GLMM, Bonferroni's test	√
Cipriano et al. (25) (Italy)	√ Analytic, observational, interventional	√ HUVEC human endothelial cells	X	X	√ PEA 10^∧^−6, 10^∧^−7, 10^∧^−8 M (*in vitro* addition)	√ 48-h alone or combined application (added to medium after Aβ administration)	√ CTRL; Aβ; Aβ+PEA 10^∧^−6+GW6471	√ Study population; experimental conditions	√ No comorbidity	√ No exclusion criteria; no confounders	√ ANOVA, Bonferroni's test	X
Brotini et al. (26) (Italy)	√ Analytic, observational, interventional	√ levodopa treated PD patients (PDSBB clinical diagnostic criteria): (a) HY scale > 0; (b) MMSE ≥26/30; (c) age>18 years; (d) levodopa therapy (eventually other PD medication) without modification over 4 consecutive weeks	√ 73 ± 8	√ Male and female	√ um-PEA 600 mg	√ Bid administration (3 months), then daily administration (9 months)	X	√ Clinical condition; age range	√ Hypertensive heart disease; Mild ischemic heart disease; Hypertension; Previous ictus cerebri; Epilepsy; History of juvenile migraine; Previous oncological surgery; Prostatic hypertrophy; Asthma; Osteoarthritis; Osteoporosis; Diabetes	√ Excluded if: (a) other forms of parkinsonism; (b) other forms of dementia; (c) unreliable patients; (d) non-compliant patients	√ GLMM, Wilcoxon signed-rank test, Bonferroni's correction, Tukey-Kramer adjusted test	X
Assogna et al. (27) (Italy)	√ Analytic, observational, interventional	√ Consecutive FTD patients (including bvFTD and PPA): 1. Age between 50 to 85 years; 2. FTLD-CDR SoB scale total score ≤ 2; 3. evidence of frontotemporal hypometabolism at PET	√ 62.35 ± 9.43	√ Male and female	√ co-ultra PEALut (um-PEA 700 mg + luteolin 70 mg) oral administration	√ Bid administration (4 weeks)	X	√ Clinical condition; age range	√ No comorbidity	√ Excluded if: (a) use of drugs modulating brain excitability in the 3 previous months; (b) other CNS NDDs; (c) psychiatric illnesses; (d) signs of concomitant CVD on MRI	√ ANOVA, Shapiro-Wilk test, Wilcoxon test, Student's *t*-test, Mauchly's test, Huynh–Feldt ε correction, Bonferroni's correction, Kruskal-Wallis non-parametric test, rmANOVA	X
Campolo et al. (28) (Italy)	√ Analytic, observational, interventional	√ Moderate TBI patients (GCS 9-13)	√ 52 ± 17.5	√ Male and female	√ co-ultra PEALut (um-PEA 700 mg + luteolin 70 mg) oral administration	√ Bid administratio*n* (180 days)	√ std	√ Clinical condition; age range	√ Diabetes; Arterial hypertension	√ Excluded if: (a) evolving to severe neurological status; (b) poor application or compliance to the study protocol; Adjusted for: (a) age; (b) educational level (Memory and cognition assessments)	√ Student's *t*-test, Mann-Whitney *U*-test, χ2 test	√

SH-SY5Y, cloned subline of a neuroblastoma cell line; co-ultra PEALut, co-ultramicronized palmitoylethanolamide and luteoline; um-PEA, ultramicronized palmitoylethanolamide; μM, micromolar; h, hours; CTRL, control; Aβ, β-amyloid precursor protein; ANOVA; analysis of variance; AD, Alzheimer's disease; ApoEε4, Apolipoprotein Eε4; Bid, twice a day; ≥, equal or more than; MMSE, Mini Mental State Examination; GLMM, Generalized linear mixed model; HUVEC, Human umbilical vein endothelial cells; PD, Parkinson's disease; PDSBB, Parkinson's Disease Society Brain Bank; HY, Hoehn and Yahr; FTD, Frontotemporal dementia; bvFTD, behavioral variant FTD; PPA, primary progressive aphasia; FTLD-CDR SoB, FTLD-modified Clinical Dementia Rating scale Sum of Boxes; ≤ , equal or less than; PET, positron emission tomography; CNS, central nervous system; NDDs, neurodegenerative disorders; CVD, cerebrovascular disease; MRI, Magnetic resonance imaging; rmANOVA, repeated-measures ANOVA; TBI, traumatic brain injury; GCS, Glasgow coma scale; std, standard.

**Table 2B:** Methodological quality of preclinical studies investigating palmitoylethanolamide and its correlations to neurocognitive disorders (NCDs).

**References**	**Study design**	**Defined study population**	**Age**	**Gender**	**PEA measure**	**Adequate PEA evaluation**	**Control**	**Comparability of subjects**	**Statistical analyses**	**Funding or sponsorship**
Scuderi et al. (29) (Italy)	√ Analytic, observational, interventional	√ Astrocytes from newborn Sprague-Dawley rats	√ PND 2	X	√ PEA 10^−7^ M (*in vitro* addition)	√ 24-hour application (added to medium after Aβ administration)	√ CTRL; Aβ	√ Study population; experimental conditions	√ ANOVA, Bonferroni's test, Newman-Keuls test	X
Benito et al. (30) (Italy)	√ Analytic, observational, interventional	√ Astrocites from newborn FAAH-KO mice; Astrocites from newborn C57/BL6 mice	√ PND 1	X	√ PEA 10 μM (*in vitro* addition)	√ 24-hour alone or combined application (added to medium prior to Aβ administration)	√ CTRL-WT; Aβ-WT	√ Study population; experimental conditions	√ ANOVA, Student's t-test, Newman-Keuls test	X
D'Agostino et al. (31) (Italy)	√ Analytic, observational, interventional	√ WT mice; PPAR-α -/- mice backcrossed to C57/BL6	X	√ Male	√ 1. First set of mice: PEA 3 mg/Kg, 10 mg/Kg, 30 mg/kg (sc administration); 2. Second and 3. Third sets of mice: 30 mg/Kg (sc administration)	√ 1. First and 3. Third sets of mice: daily administration (7 days and 5 days); 2. Second set of mice: single administration (30 min before test)	√ ScAb+VHI; Ab+VHI; Ab+GW7647	√ Study population; experimental conditions; gender	√ ANOVA, Student's t-test, Dunnett's *post hoc* test, Wilcoxon signed-rank test	√
Scuderi et al. (32) (Italy)	√ Analytic, observational, interventional	√ Sprague-Dawley rats	√ 1. Primary cultures of cerebral cortex neurons: ED 18; 2. Primary cultures of cerebral cortex astrocytes: PND 1-2	X	√ PEA 0.1 μM (*in vitro* addition)	√ Alone or combined application (added to medium after Aβ administration)	√ CTRL; Aβ	√ Study population; experimental conditions	√ ANOVA, Bonferroni's test	√
Scuderi and Steardo (33) (Italy)	√ Analytic, observational, interventional	√ Sprague-Dawley rats	√/X Primary cultures of cerebral cortex neurons: ED 18	X	√ PEA 0.1 μM (*ex vivo*/*in vivo* addition)	√ 24-h alone or combined application (added to medium after Aβ administration)	√ CTRL; Aβ	√ Study population; experimental conditions	√ ANOVA, Bonferroni's test	√
Paterniti et al. (22) (Italy)	√ Analytic, observational, interventional	√ CD1 mice	√ PND 6	X	√ co-ultra PEALut (um-PEA 0.27 μM + luteolin 0.027 μM or um-PEA 2.7 μM + luteolin 0.27 μM or um-PEA 27 μM + luteolin 2.7 μM) addition to medium	√ Single application (added to medium after 21-day incubation, 2 h before Aβ)	√ CTRL; Aβ	√ Study population; age; experimental conditions	√ ANOVA, Bonferroni's test	X
Scuderi et al. (34) (Italy)	√ Analytic, observational, interventional	√ Sprague-Dawley rats	√/X Adult	√ Male	√ PEA 10 mg/Kg (ip administration)	√ Daily administration (7 days)	√ VHI; VHI+Aβ	√ Study population; experimental conditions; gender; age	√ ANOVA, Bonferroni's test	√
Cipriano et al. (25) (Italy)	√ Analytic, observational, interventional	√ C6 rat glioma cells	X	X	√ PEA 10^∧^−6, 10^∧^−7, 10^∧^−8 M (*in vitro* addition)	√ 48-h alone or combined application (added to medium after Aβ administration)	√ CTRL; Aβ; Aβ+PEA10^∧^−6+GW6471	√ Study population; experimental conditions	√ ANOVA, Bonferroni's test	X
Tomasini et al. (35) (Italy)	√ Analytic, observational, interventional	√ 3 × Tg-AD mice; non-Tg mice	X	X	√ PEA 0.1 μM (*in vitro* addition)	√ 24-h application (added to medium 1 h before Aβ administration)	√ (3xTg-AD, non-Tg): CTRL; Aβ	√ Study population; experimental conditions	√ ANOVA, Newman-Keuls test	√
Caltagirone et al. (24) (Italy)	√ Analytic, observational, interventional	√ Wistar rats	X	√ Male	√ co-ultraPEALut 1 mg/Kg (oral administration)	√ Double administration (1 h after ischemia, 6 h after reperfusion)	√ MCAo+VHI; sham+VHI	√ Study population; experimental conditions; gender	√ ANOVA, Student's *t*-test, Bonferroni's test, Newman-Keuls test	√
Siracusa et al. (36) (Italy)	√ Analytic, observational, interventional	√ CD1 mice; Sprague-Dawley rats	X	√ Male	√ 1. Brain tissue levels (healthy rats); 2. co-ultra PEALut 1 mg/Kg (oral administration) (mice)	√ 1. Single assessment (healthy rats); 2. Daily administration (15 days, 24 h after VaD induction) (mice)	√ sham+VHI; sham+PEA; VaD+VHI	√ Study population; gender; experimental conditions	√ ANOVA, Bonferroni's test	X
Beggiato et al. (37) (Italy)	√ Analytic, observational, interventional	√ Cerebral cortex astrocytes from C57/BL6 mice; Cerebral cortex neurons from C57/BL6 mice	√ 1. Primary cultures of cerebral cortex neurons: ED 18; 2. Primary cultures of cerebral cortex astrocytes: PND 1-2	X	√ PEA 0.1 μM (*in vitro* addition)	√ 24-h alone or combined application (added to medium 1 h before Aβ administration)	√ CTRL; Aβ	√ Study population; experimental conditions	√ ANOVA, Newman-Keuls test	√
Bronzuoli et al. (20) (Italy)	√ Analytic, observational, interventional	√ 1. 3 × Tg-AD mice; 2. non-Tg mice	√ 1. *In vivo*: 3 months; 2. *In vitro*: PND 1-2	√ Male	√ 1. um-PEA 10 mg/kg (sc administration); 2. PEA 0.01, 0.1, 1 μM (*in vitro* addition)	√ 1. Daily administration (90 days); 2. 24-h application (added to medium after 7 and 28 days, for astrocytes and neurons respectively)	√ (3xTg-AD, non-Tg): CTRL	√ Study population; experimental conditions; gender; age	√ ANOVA, Student's *t*-test, Bonferroni's test	X
Crupi et al. (39) (Italy)	√ Analytic, observational, interventional	√ CD1 mice	√ 21 months	√ Male	√ PEAm 10 mg/Kg (oral administration)	√ Daily administration (60 days)	√ sham+VHI; sham+PEA; MPTP+VHI	√ Study population; age; gender; experimental conditions	√ ANOVA, Bonferroni's test	X
Scuderi et al. (20) (Italy)	√ Analytic, observational, interventional	√ 3 × Tg-AD mice	√ 1. First set of mice: 3 to 6 months; 2. Second set of mice: 9 to 12 months	√ Male	√ um-PEA 28 mg (sc administration)	√ Daily administration (3 months)	√ non-Tg mice; placebo	√ Study population; age; gender; experimental conditions	√ ANOVA, Tukey's HSD test, Bonferroni's test	√
Boccella et al. (40) (Italy)	√ Analytic, observational, interventional	√ C57/BL6 mice	X	√ Male	√ um-PEA 10 mg/Kg (ip administration)	√ Daily administration (15 days, starting 15 days after sham or SNI)	√ sham groups; SNI+VHI; SNI+MPEP; SNI+MPEP+PEA; SNI+MDCPG; SNI+MDCPG+PEA	√ Study population; gender; experimental conditions	√ ANOVA, Dunnett's multiple comparison *post hoc* test, Student's t-test, Bonferroni's test	√
Boccella et al. (41) (Italy)	√ Analytic, observational, interventional	√ WT mice; PPAR-α -/- mice backcrossed to C57/BL6	X	√ Male	√ 1. um-PEA 10 mg/Kg (ip administration); 2. Brain tissue levels	√ 1. Daily administration (15 days, starting 15 days after sham or SNI); 2. Single assessment	√ sham+VHI; sham+PEA; SNI+VHI	√ Study population; gender; experimental conditions	√ ANOVA, Dunnett's multiple comparison *post hoc* test, Student's *t*-test, D'Agostino-Pearson's normality test, Bonferroni's test, Kruskall-Wallis test, Dunn's test	√
Impellizzeri et al. (42) (Italy)	√ Analytic, observational, interventional	√ CD1 mice	X	√ Male	√ 1. PEA-OXA 10 mg/kg (oral administration); 2. Brain tissue levels	√ 1. Daily administration (15 days); 2. Single assessment	√ sham+VHI; sham+PEA; VaD+VHI	√ Study population; gender; experimental conditions	√ ANOVA, Bonferroni's test, Neuman-Keuls multiple comparison test	X
Piscitelli et al. (43) (Italy)	√ Analytic, observational	√ Tg2576 mice	√ 4–15 months	√ Male	√ 1. Plasma levels; 2. Brain tissue levels	√ Multiple assessment (T1 presymptomatic: 4–6 months; T2 mild symptomatic: 7–10 months; T3 symptomatic: 12–15 months)	√ WT	√ Age; experimental conditions	√ ANOVA, Tukey's *post hoc* test, Tukey HSD test	√
Zimmermann et al. (44) (Germany)	√ Analytic, observational	√ NEX-Cre mice (C57/BL6 background)	√ 2–3 months	√ Male	√ Brain tissue levels	√ Single assessment	√ AAV-WT; AAV-Glu-empty	√ Age; gender; experimental condition	√ ANOVA, Tukey's *post hoc* test, Student's *t*-test, Kolmogorov-Smirnov test, Bonferroni's test, Sidak's multiple comparison test	√
Beggiato et al. (45) (Italy)	√ Analytic, observational, interventional	√ 3 × Tg-AD mice	√ 1. Primary cultures of cerebral cortex neurons: ED 18; 2. Primary cultures of cerebral cortex astrocytes: PND 1-2	X	√ PEA 0.1 μM (*in vitro* addition)	√ 24-h application (added to medium 1 h before Aβ)	√ non-Tg mice; 3xTg-AD(CTRL); 3xTg-AD(Aβ)	√ Study population; experimental conditions	√ Student's *t*-test	√
Beggiato et al. (46) (Italy)	√ Analytic, observational, interventional	√ 3 × Tg-AD mice; C57BL6/129SvJ mice	√ 2 months ± 2 weeks of age	√ Male	√ 1. um-PEA 100 mg/Kg (oral administration); 2. Brain tissue levels; 3. Plasma levels	√ 1. Pharmacokinetic studies: (a) single or daily (8 days) administration; (b) single brain tissue or plasma assessment (prior to PEA; 1, 1.5, 3, 4 h after PEA); 2. Biobehavioral studies: daily administration (3 months)	√ non-Tg; 3xTg-AD+VHI	√ Age; gender; experimental conditions	√ ANOVA, Tukey's HSD test, Bonferroni's test, Student's *t*-test	√
Facchinetti et al. (47) (Italy)	√ Analytic, observational, interventional	√ Sprague-Dawley rats	√/X Adult rats	√ Male	√ co-ultra PEALut 5 mg/Kg (ip administration)	√ Daily administration (14 days)	√ VHI; VHI(Aβ)	√ Study population; age; gender; experimental conditions	√ ANOVA, Bonferroni's test	√
Lama et al. (48) (Italy)	√ Analytic, observational, interventional	√ C57/BL6 mice	√ 6 weeks	√ Male	√ um-PEA 30 mg/Kg (oral administration)	√ Daily administration (7 weeks)	√ STD; HFD	√ Study population; age; gender; experimental conditions	√ ANOVA, Bonferroni's test	X
Boccella et al. (49) (Italy)	√ Analytic, observational, interventional	√ C57/BL6 mice	√ 4-5 weeks	√ Male	√ PEA-OXA 10 mg/kg (ip administration)	√ Daily administration (16 days, starting 14 days after SNI or sham surgery)	√ sham+VHI; sham+PEA; SNI+VHI	√ Study population; age; gender; experimental conditions	√ ANOVA, Kolmogorov–Smirnov test	√
Campolo et al. (21) (Italy)	√ Analytic, observational, interventional	√ CD1 mice	√ 10–12 weeks	√ Male	√ co-ultra PEALut 1 mg/Kg (oral administration)	√ Daily administration (72 h and 7 days, 1 h after craniotomy)	√ sham; TBI	√ Study population; age; gender; experimental conditions	√ Student's *t*-test, Mann-Whitney *U*-test, χ2 test	X
D'Antongiovanni et al. (50) (Italy)	√ Analytic, observational, interventional	√ SAMP8 mice	√ 4 months	X	√ 1. PEA 5 mg/Kg (oral administration) 2. PEA 0.1 μM (*in vitro* addition)	√ 1. Daily administration (2 months); 2. 1-h application (added to medium 4 h after LPS, 1 h before Aβ)	√ SAMR1; SAMP8; CTRL; LPS+Aβ	√ Study population; age; experimental conditions	√ ANOVA, Tukey's test, Student's *t*-test	√
Gaspar et al. (51) (Ireland)	√ Analytic, observational, interventional	√ Sprague-Dawley rats	X	√ Male	√ 1. PEA 2 mg/Kg (ip administration) 2. Brain tissue levels	√ 1. Single administration (day 28 post-CFA); 2. Single assessment	√ noCFA groups; CFA+VHI; CFA+GSK; CFA+GW6471; CFA+GW9662	√ Study population; gender; experimental conditions	√ ANOVA, SNK *post hoc* test, Cohen's d coefficient, Kruskal Wallis test, Friedman's test, Dunn's *post hoc* test, Mann-Whitney *U*-test, Bonferroni's test, Shapiro-Wilk test, Levene's test	√
Gatta et al. (52) (Italy)	√ Analytic, observational, interventional	√ 1. *In vitro* experiment: BV2 microglial cell model; 2. *Ex vivo* experiment: C57/BL6 mice	√/X *Ex vivo* primary cultures of cerebral cortex microglia: PND 3	X	√ PEA 10 μM (*in vitro* addition)	√ 24-/48-hour application (added to medium before or in presence of LPS or Aβ)	√ CTL; LPS+PEA; Aβ+PEA	√ Study population; experimental conditions	√ ANOVA, Tukey's test	√

PND, postnatal day; PEA, palmitoylethanolamide; M, molar; Aβ, β-amyloid precursor protein; CTRL, control; FAAH, Fatty acid amide hydrolase; KO, Knock-out; μM, micromolar; WT, Wild-type; PPAR, Peroxisome proliferator-activated receptor; C57/BL6, multipurpose mouse model; mg/Kg, milligrams per kilogram; sc, subcutaneous; ScAb, Scrambled Ab25-35 peptide; VHI, vehicle; Ab, Ab25-35 peptide; GW7647, PPARα agonist; ED, embryonic day; CD1, multipurpose mouse model; co-ultra PEALut, co-ultramicronized palmitoylethanolamide and luteoline; um-PEA, ultramicronized palmitoylethanolamide; ip, intraperitoneal; C6, glial cell line; GW6471, PPARα antagonist; 3xTg-AD, triple-transgenic mouse model of AD; non-Tg, non-transgenic mouse model; h, hours; MCAo, middle cerebral artery occlusion; VaD, vascular dementia; PEAm, micronized PEA; MPTP, 1-methyl-4-phenyl-1,2,3,6-tetrahydropyridine; HSD, honestly significant difference; SNI, spare nerve injury; MPEP, 2-Methyl-6-(phenylethynyl) pyridine; MDCPG, (RS)-4-(1-amino-1-carboxyethyl)phthalic acid; PEA-OXA, N-Palmitoylethanolamine-oxazoline; Tg2576, transgenic mouse model; T(1, 2, 3), time (1, 2, 3); NEX-Cre, mouse line expressing Cre recombinase under control of regulatory sequences of NEX; AAV, adeno-associated virus; Glu, glutamatergic neurons; 129SvJ, multipurpose mouse model; STD, standard-diet group; HFD, high-fat diet; TBI, traumatic brain injury; SAMP8, Senescence Accelerated Mouse-prone 8; SAMR1, Senescence-Accelerated Mouse-Resistant 1; LPS, lipopolysaccharide; CFA, Complete Freund's Adjuvant; GW9662, PPARγ antagonist; GSK, GSK0660 (PPARβ/δ antagonist); BV2, microglial cell line.

### Statistical analysis

When deemed appropriate, studies with similar methodologies and output measures were gathered to be further explored from a meta-analytic perspective. Specifically, baseline and post-treatment values were extracted. Change-from-baseline standard deviation was calculated when not reported by assuming a moderate pre-post correlation coefficient (r = 0.7) as suggested by Cochrane Handbook 5.1 (https://handbook-5-1.cochrane.org/chapter_16/16_1_3_2_imputing_standard_deviations_for_changes_from_baseline.htm). Data were pooled by using a DerSimonian and Laird random-effects model (55). A meta-regression model was developed to investigate the effects of patients' age and length of follow up. I-squared index was calculated to assess heterogeneity among studies. Publication bias was not investigated due to the low number of studies included. Data were analyzed by the statistical software STATA software, version 16.1.

## Results

### Identified studies for inclusion in systematic review

**Figure 1:**
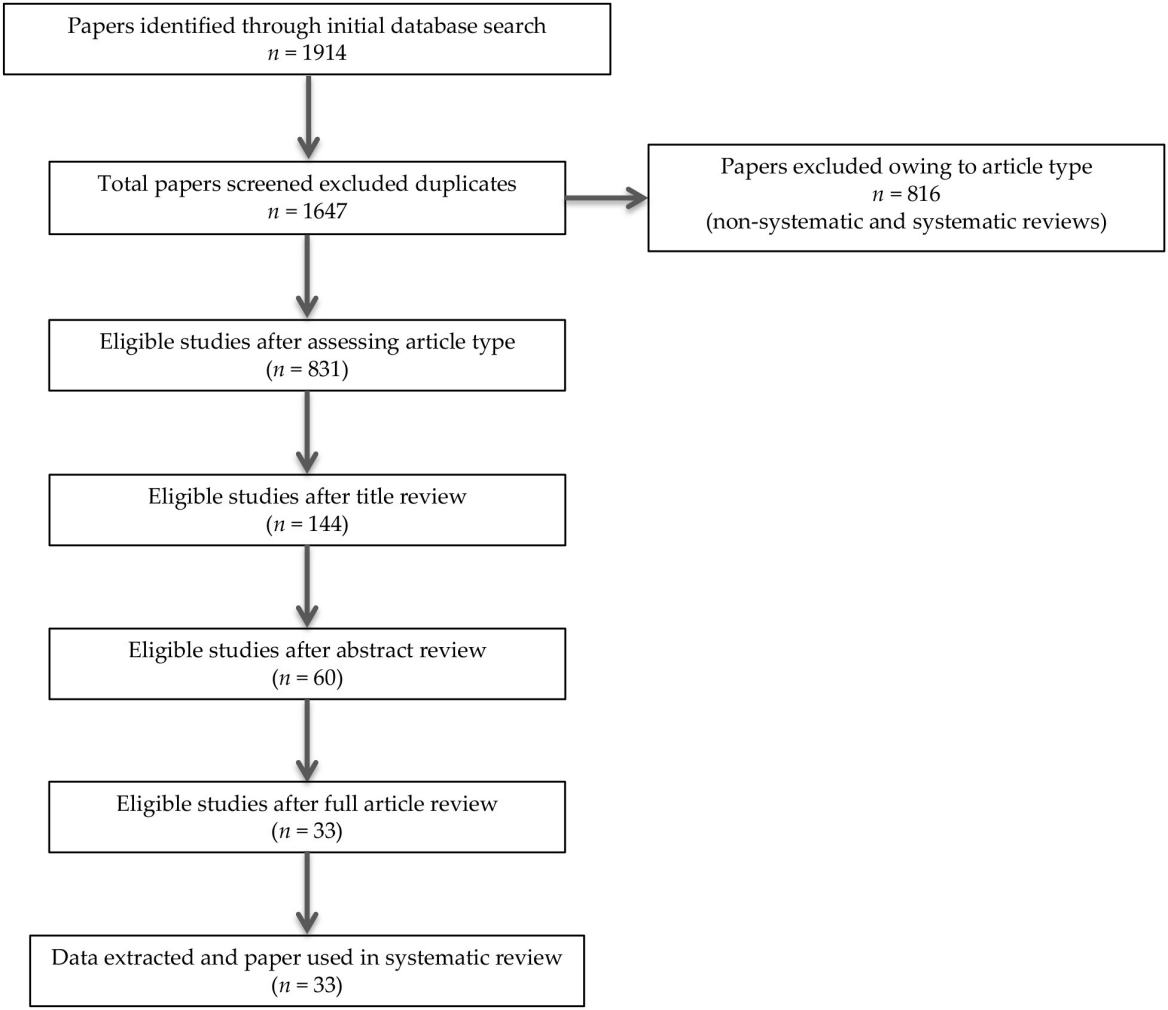
PRISMA flowchart of search strategy for systematic review.

In summary, 1914 records were identified through the initial data search. After excluding duplicates as well as articles owing to article type (systematic and non-systematic reviews), by using a three-step screening approach, titles, abstracts, or full texts of all records were screened against the inclusion and exclusion criteria (Figure 1). A final list of thirty-three studies was used for systematic analysis in this review, including 4 studies conducted only in human populations, 26 studies performed only in animal models, and 3 studies including both animal and human data, investigating different aspects of the palmitoylethanolamide (PEA) signaling pathway (Table 1). These include (i) *in vivo* PEA treatment exposure in humans with different neurocognitive disorders (NCDs) and related conditions (4 studies; Table 1A); (ii) *in vitro* PEA exposure in Amyloid-β (Aβ) exposed human cells (2 studies; Table 1A); (iii) PEA quantitative blood assessment in humans with Alzheimer's Disease (AD; 1 study; Table 1A); (iv) *in vivo* PEA exposure in animal models of NCDs and related conditions (17 studies; Table 1B); (v) *in vitro* PEA exposure in Aβ exposed animal cells (7 studies; Table 1B) and AD animal model cells (4 studies; Table 1B); (vi) *ex vivo* PEA exposure in in Aβ exposed animal cells (3 studies; Table 1B) and AD animal model cells (1 study; Table 1B); and (vii) PEA quantitative brain/tissue assessment in animal models of different NCDs (7 studies; Table 1B). Additional data on methodological quality of studies conducted in humans and animals are reported in Tables 2A,B. A detailed presentation of human and animal results is reported in Supplementary Tables 1A,B. A brief synthesis of the main results is presented below.

### *In vivo* PEA treatment exposure in humans with different NCDs and related conditions

Most human studies identified in this review addressed the effects of PEA exposure on cognitive function (Table 1A), using similar but not overlapping methodologies (Table 2A) in terms of disorder [stroke (24), Parkinson's disease (PD) (26), Frontotemporal dementia (FTD) (27), and traumatic brain injury (TBI) (28)], PEA dosage [600 mg bid/daily (26), 700 mg bid (24, 27, 28)], PEA formulation [alone (26), with luteolin (24, 27, 28)], PEA mode of administration [sublingual (24, 26), oral administration (27, 28)], and PEA period of exposure [4 weeks (27), 60 days (24), 180 days (28), 12 months (26)]. Apart from a single study that adopted a placebo-controlled design (28), all studies lacked a controlled condition (24, 26, 27). Nevertheless, results indicated a beneficial effect of PEA in ameliorating cognitive impairment following cerebral ischemia (24) and TBI (28) as well as non-motor aspects of experiences of daily living (nM-EDL; e.g., anxious-depressive symptoms, sleep problems, and fatigue) in PD (26) and frontal lobe disfunctions and behavioral disturbances in FTD (27). Noteworthy, PEA was well tolerated, in the absence of any relevant side effect across all the studies, and for the entire duration of the compound administration.

**Figure 2:**
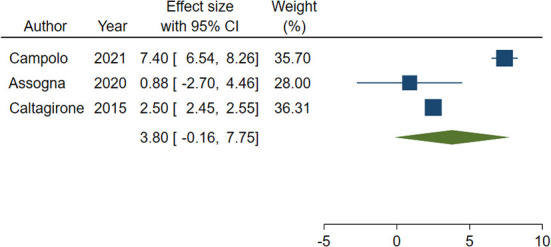
Forest plot showing the pooled Mini-Mental State Examination (MMSE) change from baseline. Homogeneity (I-squared): 98.40%, *p* < 0.001; Estimation by DerSimonian and Laird random-effects model.

Out of the 4 studies, 3 (24, 27, 28) adopted the same tool to investigate impairment of cognitive abilities, the Mini Mental State Examination (MMSE), a widely accepted instrument to gather the cognitive state of patients suffering from NCDs (56), and were included in the meta-analysis. A total of 282 patients were considered. Weighted mean and standard deviation were 69.70 and 12.51 years respectively. The pooled change-from baseline was 3.80 points (95% C.I. −0.16–7.75; Figure 2). Meta-regression did not show a significant effect of patients' age (coefficient: 0.17; 95% CI: −0.28–0.61) or length of follow-up (coefficient: 0.06; 95% CI: −0.01–0.12). The heterogeneity was remarkable (I-squared: 98.40%, *p* < 0.001).

### *In vitro* PEA exposure in amyloid-β (Aβ) exposed human cells

In total, two studies did not evaluate the effect of PEA administration in humans suffering from NCDs and related conditions, while analyzing the biological effect of PEA in human cells from *in vitro* models of NCDs (22, 25). In such studies, AD features were induced by Aβ stimulation in either neuron-like cells (22) or Human Umbilical Vein Endothelial Cells (HUVEC) (25) (Tables 1A, 2A). When compared to control conditions, PEA alone (25) or combined with luteolin (22) was found to blunt Aβ-induced astrocyte activation and exert protective effects on glial cells (22) possibly via a peroxisome proliferator-activated receptor alpha (PPAR-α)-mediated reduction of the production of pro-inflammatory and pro-angiogenic markers (25).

### PEA quantitative blood assessment in humans with Alzheimer's disease

This systematic review identified a single study specifically analyzing peripheral PEA levels in humans suffering from AD, as compared to healthy controls (23) (Tables 1A, 2A). Despite not significantly different between AD patients and controls, PEA levels appeared to be associated with cognitive performance among patients. Interestingly, 2-Arachidonoylglycerol (2-AG) levels also correlated with memory, attention, and underlying brain atrophy, suggesting an extensive role of the endocannabinoid system in the neuropathology of AD (23).

### *In vivo* PEA exposure in animal models of NCDs and related conditions

Most evidence regarding a potential therapeutic effect of PEA in NCDs was gathered from preclinical studies administering the compound to animal models of NCDs (Table 1B), including AD (20, 31, 38, 46, 47, 50), Aβ-exposed (34), middle cerebral artery occlusion (MCAo) (24), PD (39), vascular dementia (VaD) (36, 42), spared nerve injury (SNI) (40, 41, 49), TBI (28), Complete Freund's Adjuvant (CFA) (51), and high-fat diet (48) models. Despite the evidence of similar methodologies across the reviewed studies, a certain heterogeneity was found in terms of animal type [mice (20, 28, 31, 36, 38–42, 46, 48–50), rat (24, 34, 47, 51)], period of exposure [from 4 to 5 weeks old to 21 months old), PEA formulation [alone unspecified, PEA (31, 34, 50, 51); alone micronized, PEAm (39); alone ultra-micronized, um-PEA (20, 38, 40, 41, 46, 48); combined with oxazoline, PEA-OXA (42, 49); combined with luteolin, PEALut (24, 28, 36, 47)], PEA mode of administration [intraperitoneal (34, 40, 41, 47, 49, 51), subcutaneous (20, 31, 38), oral (24, 28, 36, 39, 42, 46, 48, 50)], dosage of PEA [2 to 10 mg/kg for intraperitoneal administration (34, 40, 41, 47, 49, 51), 3 to 30 mg/kg for subcutaneous (20, 31, 38), 1 to 100 mg/kg for oral administration (24, 28, 36, 39, 42, 46, 48, 50)], and duration of exposure (from single administration to 3 months) (Table 2B).

Studies conducted in experimental models of AD found a dose-dependent (31), early intervention (20), and chronic (46) effect of PEA in reducing highly representative features of AD such as working memory-like impairments (31) and learning and memory deficits (20, 31, 46) as well as the associated depressive-like and anhedonia-like phenotypes (20). Such effect was dependent of PPAR-α activation (31) and related to the ability of PEA of reducing AD-associated biomolecular mechanisms such as Aβ expression (20), abnormal hippocampal tau phosphorylation (20), lipid peroxidation (31), protein nytrosylation (31), inducible nitric oxide synthase induction (31) and reactive oxygen species production (46), and caspase3 activation (31). A role of PEA administration in restoring astrocyte (20, 38, 47) and glutamatergic (20, 46) functions, restraining neuroinflammation (20, 46, 47) and enteric inflammation and motor dysfunction (50), and promoting neuronal viability (38), hippocampal neuronal survival (47), and microtubule-associated protein 2 (MAP2) expression (20), in AD mice, was also found.

Similar findings were found among Aβ-exposed animals (Tables 1B, 2B) where PEA reduced memory impairments, reactive gliosis, amyloidogenesis, and neuroinflammation, and improved neuronal integrity, *via* PPAR-α activation (34). The same remarks suggesting an effect of PEA in rescuing memory deficits and injured hippocampal neurons, possibly by exerting anti-inflammatory and neuroprotective effects, were reported in preclinical models of VaD (36, 42). Finally, converging evidence for an improving effect of PEA administration on learning and memory and their biological underpinnings was also found in the context of brain ischemia reperfusion injury (24), PD (39), SNI (40, 41, 49), TBI (28), CFA (51), and high-fat diet (48), where cognitive decline is a common complication of the disease.

### *In vitro* PEA exposure in Aβ exposed animal cells and AD animal model cells

In total, 11 studies evaluated the effect of *in vitro* PEA exposure on several neurobiological mechanisms underlying NCDs (Tables 1B, 2B), using both Aβ exposed animal cells (25, 29, 30, 32, 33, 35, 37) and AD animal model cells (38, 45, 50, 52). PEA application was reported to reduce Aβ-induced neuroinflammation and astrocyte activation (25, 29, 30, 32, 33, 37) as well as angiogenesis (25), exerting a protective effect on neuronal cells (32, 33, 37). Such effect was dependent on PPAR-α and cannabinoid receptor type 1 (CB1) and 2 (CB2) activation (29). Also, PEA was found to exert protective properties in wild-type mouse cell cultures but not in AD mouse neuronal cultured cells overexpressing Aβ, suggesting its effectiveness in early AD or when Aβ is accumulating and initiating damage in the central nervous system (35). Similar findings were found among AD models where *in vitro* PEA application reduced neuroinflammation (52) and astrogliosis (38, 45), supporting neuronal viability and survival (38, 45), and also improving enteric inflammation (50).

### *Ex vivo* PEA exposure in in Aβ exposed animal cells and AD animal model cells

To confirm the results obtained with the *in vitro* models, some studies made the same PEA treatment *ex vivo* (Tables 1B, 2B) with organotypic cultures challenged with Aβ (22, 32, 33) or lipopolysaccharide (LPS) (52). Converging evidence suggests that PEA may enhance neuroprotection against the neurodegenerative processes associated with Aβ deposition, including astrocyte activation (32, 33) and neuroinflammation (32, 33, 52). Also, PEA exposure showed specific effects in reducing inducible nitric oxide synthase (22), glial fibrillary acidic protein expression (22), and apoptosis (22, 52) as well as restoring neuronal nitric oxide synthase (22) and brain derived neurotrophic factor (BDNF) (22).

### PEA quantitative brain/tissue assessment in animal models of different NCDs

Seven studies analyzed PEA levels in the brain and tissues of animal models of NCDs (Tables 1B, 2B), including VaD (36, 42), SNI (41), CFA (51) and genic models of AD (43, 46) and related conditions (44). Genetically inducing a reduction in PEA levels resulted in changes in hippocampal synaptic activity and aberrant cognition (44). Consistently, PEA levels were reported to be reduced after VAD induction (42), but restored following exogenous PEA administration (36, 42), possibly accounting for the observed therapeutic effects (36, 42). Similarly, plasma and brain PEA levels were found to be slightly lower in a genic model of AD, despite not significantly (46). Another study in a genic model of AD revealed that changes in PEA levels may depend on the disease stage, from being relatively higher in the pre-symptomatic and mild symptomatic phases to being relatively lower in the symptomatic stage (43). Further, no differences in PEA levels were observed in CFA-injected models (51), neither PEA administration affected PEA levels in SNI models (41), warranting further investigation of a potential selective effect of PEA in primary cognitive decline.

## Discussion

This is the first systematic review of all studies investigating the biobehavioral effects of palmitoylethanolamide (PEA) with reference to cognitive decline, that is the core symptomatologic domain of neurocognitive disorders (NCDs) (4). Independently of potential effects of PEA on additional features of NCDs, such as motor impairments, pain, and overall disability [recently reviewed here (57)], disentangling whether PEA is effective in improving cognition, possibly corroborated by evidence of a restoring effect on its neurobiological underpinnings, is of paramount importance to tip the scales toward considering PEA an adjunctive therapeutic option for NCDs. Based on evidence that degeneration of basal forebrain neurons causes a loss of cholinergic tone in the basal forebrain cholinergic system, with implications for the development of cognitive decline (58), most research has focused on the role of acetylcholinesterase (AChE) inhibitors as a potential treatment for NCDs (59). Further, in the absence of other successful interventions, recent research is focusing on the possibility to refine AChE inhibitors to maximize their potential (10). However, growing evidence indicates a crucial role for neuroinflammation in neurodegeneration (8) and a potential therapeutic effect of neuroinflammation modulation in contrasting neurodegeneration at both the neurobiological and behavioral level (11). In this regard, recent research highlights the importance that cannabinoid-related compounds, whose actions depend on the interaction with non-CB receptors, may have in terms of anti-inflammatory properties (16–18), in turn accounting for their ability to mitigate biological mechanisms involved in neurodevelopmental disorders (53), epilepsy (15), and neurodegeneration (12).

Overall, this review indicated that PEA, whose biological effects are related to indirect activation of CB1 receptors as well as PPAR-α and Transient Receptor Potential Vanilloid 1 (TRPV1) modulation (14, 60), may be involved in NCDs and related conditions. With reference to human studies, evidence was obtained from interventional studies of the positive cognitive effects of PEA supplementation in humans, benefits of PEA at the neurobiological level in both *in vivo* and *in vitro* human studies, and a single observational study that changes in the PEA tone affect cognitive performance. Regarding animal studies, evidence was obtained from interventional studies of a PPAR-α-dependent, dose-dependent, and early intervention pro-cognitive effect of PEA, benefits of PEA on several biomolecular mechanisms in *in vivo, in vitro*, and *ex vivo* studies, and observational studies that a reduction in the PEA tone affects cognitive performance and related hippocampal activity, possibly specific to primary cognitive decline in the symptomatic stage.

Some important findings from this systematic review deserve to be highlighted. First, NCDs represent the group of conditions where the use of PEA seems to be the most supported by research studies, with an overwhelming convergence of evidence toward a therapeutic effect on core cognitive symptoms and underlying neurobiological underpinnings. Also, compared to other conditions, such as autism spectrum disorders (53) or epilepsy (15), where the evidence for a therapeutic potential of PEA is robust, but very limited (53) or absent (15) in humans, the present review identified 7 studies performed in humans. Such studies were either *in vivo, in vitro*, or observational studies, and a preliminary meta-analysis of studies assessing cognition before and after PEA administration (24, 27, 28) revealed an effect of PEA in partially reversing cognitive decline. Instead, studies of PEA in other neuropsychiatric disorders are still in their infancy, despite results seem promising. For instance, very recent clinical trials provided initial evidence that PEA may be a valid adjunctive treatment in acute mania (61) and schizophrenia (62). It is worth mentioning that, while not being the focus of this review, results presented here support a potential role of PEA also in depressive-like symptoms (20, 26).

Second, thanks to numerous preclinical studies, performed adopting different methodological strategies, as well as some *in vitro* human studies and an observational human study, this review was able to offer a sufficiently solid neurobiological explanation for the therapeutic effects of PEA. In fact, PEA was found to control Aβ expression (20), hippocampal tau phosphorylation (20), and associated astrocyte/glial dysfunction (20, 22, 25, 29, 30, 32, 33, 37, 38, 45, 47), resulting in increased neuronal viability (38, 45) and survival (22, 38, 45, 47, 52), MAP2 (20) and BDNF (22) expression, and overall neuroprotection (32, 33, 36, 37, 42). An effect of PEA in controlling glutamatergic function (20, 46) as well as neuroinflammation (20, 25, 32, 33, 36, 42, 46, 47, 52) and enteric inflammation (50) was observed, with specific modulation of lipid peroxidation (31), protein nytrosylation (31), inducible nitric oxide synthase induction (22, 31) and reactive oxygen species production (46), and caspase3 activation (31). PEA effects seemed to depend on PPAR-α activation (25, 34).

Third, brain PEA levels were found to be reduced in preclinical models of primary NCDs, that is VAD (42) and AD (46), the latter possibly as a result of the disease progression (43), further corroborating a potential need for its supplementation. Consistently, PEA level restoration *via* supplementation seemed to explain the therapeutic effect observed in VAD (36, 42). Instead, less clear appeared the role of PEA levels in other models of NCDs (41, 51). However, even when not different from a control group, animal brain (44) and human blood (23) PEA levels were found to be associated with modulation of cognitive function.

Fourth, some information was gathered in terms of PEA dosages and therapeutic window. Specifically, a a dose-dependent effect of PEA was revealed (31), with PEA exerting its maximal potential in the early stages of NCDs (20, 35). Interestingly, this may be due to PEA levels being still high in the early stages of NCDs, possibly reflecting a compensatory innate mechanism, before falling in the frank symptomatic stage (43).

Finally, the effect of PEA did not seem to be confined to the so-called primary dementias, such as AD or VAD. In fact, PEA ameliorated cognitive domains and associated symptoms also in patients with stroke (24), Parkinson's disease (PD) (26), and traumatic brain injury (TBI) (28), as well as learning and memory and underlying neurobiology in animal models of brain ischemia reperfusion injury (24), PD (39), spared nerve injury SNI (40, 41, 49), TBI (28), Complete Freund's Adjuvant (CFA) (51), and high-fat diet (48). Nevertheless, as for instance PEA levels were not altered in CFA-injected models (51), neither SNI models (41), further studies are needed to investigate potential differential mechanisms of action for PEA in primary vs. other NCDs.

The findings of this systematic review must be seen considering some strengths and limitations. Research in the field is quite advanced, even though especially in animal models. Despite being tested in different conditions, PEA effect needs to be further studied to fully address its relevance for the different clinical phenotypes of cognitive decline. In other words, evidence does not fully clarify whether PEA is useful only in primary cognitive decline associated with NCDs (e.g., AD) or also secondary (e.g., PD) and acquired (e.g., TBI) ones. While the beneficial effects of PEA in NCDs seem to be reasonably mediated by a protective role of the compound against altered neuroinflammation and related mechanisms, whether such effect is sustained in the longer-term remains to be tested. Longer-term studies are needed to support a potential effect of PEA as a disease modifying drug in blunting or halting the NCD course. Also, PEA levels seem to be altered in NCDs and differently depending on the phase of illness. However, whether this can be considered a biomarker for diagnosis and treatment response deserves additional studies. Finally, no study made a direct comparison of different PEA formulations in the same population, making it difficult to draw any conclusion about the potential superiority of any of such pharmaceutical forms.

In conclusion, this review revealed several experimental and observational investigations of PEA and its pathway in NCDs. Evidence discussed here converges in reporting alterations of the PEA signaling, implications for NCD-related biobehavioral manifestations, and benefits from PEA supplementation. In particular, PEA seems to be therapeutic in improving cognitive performance, whose decline is a characteristic manifestation of NCDs. Importantly, no serious adverse effects were reported across the *in vivo* PEA treatment exposure human studies, suggesting that PEA supplementation may represent not only an effective treatment strategy in NCDs but also exempt from major health risks.

## Data availability statement

The original contributions presented in the study are included in the article/Supplementary material, further inquiries can be directed to the corresponding author.

## Author contributions

MC, RB, CC, EB, DP, EM, GD, and MB: conceptualization, methodology, resources, validation, and writing–review and editing. MC, RB, CC, EB, DP, and GD: data curation, investigation, and visualization. GD: formal analysis. MC and MB: project administration. MC: supervision. MC, RB, CC, and GD: writing–original draft preparation. All authors have read and agreed to the published version of the manuscript.

## Conflict of interest

Author MC has been a consultant/advisor to GW Pharma Limited, GW Pharma Italy SRL and F. Hoffmann-La Roche Limited, outside of this work. The remaining authors declare that the research was conducted in the absence of any commercial or financial relationships that could be construed as a potential conflict of interest.

## Publisher's note

All claims expressed in this article are solely those of the authors and do not necessarily represent those of their affiliated organizations, or those of the publisher, the editors and the reviewers. Any product that may be evaluated in this article, or claim that may be made by its manufacturer, is not guaranteed or endorsed by the publisher.

## Supplementary material

Supplementary Table 1A Results of clinical studies investigating palmitoylethanolamide and its correlations to neurocognitive disorders (NCDs). IκBα, nuclear factor of kappa light polypeptide gene enhancer in B-cells inhibitor, alpha; Aβ, β-amyloid precursor protein; < , lower than; CTRL, control; PEA, palmitoylethanolamide; >, higher than; NFκB, nuclear factor kappa-light-chain-enhancer of activated B cells; μM, micromolar; vs., compared to; NS, not significant; 2-AG, 2-arachidonoylglycerol; AD, Alzheimer's disease; AEA, anandamide; OEA, oleoylethanolamide; ↓, reduced; ↑, increased; CNS, Canadian Neurological Scale; T0, baseline; T30, after 30 days; MMSE, Mini Mental State Examination; p-p38, phospho-p38; MAPK, Mitogen-activated protein kinase; VEGF, Vascular endothelial growth factor; GW6471, PPARα antagonist; HUVEC, Human umbilical vein endothelial cells; ATP, Adenosine triphosphate; nM-EDL, Non-Motor Aspects of Experiences of Daily Living; NPI, Neuropsychiatric Inventory; FAB, Frontal Assessment Battery; SAND, Screening for Aphasia in Neurodegeneration; LICI, long-interval intracortical inhibition; ISI, inter-stimulus-interval; SICI, short-interval intracortical inhibition; ICF, intracortical facilitation; SAI, short-latency afferent inhibition; LTP, long-term potentiation; std, standard; BNCE, Brief Neuropsychological Cognitive Examination; BDI, Beck's inventory depression scale. Bold font emphasizes significant results.

Supplementary Table 1B Results of preclinical studies investigating palmitoylethanolamide and its correlations to neurocognitive disorders (NCDs). GFAP, Glial fibrillary acidic protein; S100B, S100 calcium-binding protein B; Aβ, β-amyloid precursor protein; >, higher than; CTRL, control; PEA, palmitoylethanolamide; GW9662, PPARγ antagonist; < , lower than; MK, MK886 (PPARα antagonist); vs., compared to; NS, not significant; iNOS, inducible nitric oxide synthase; COX-2; cyclooxygenase-2; PGE2, Prostaglandin E2; NO–, Nitrogen Dioxide; TNF-α, tumor necrosis factor alpha; IL-1β, Interleukin 1 beta; p-p38, pospho-p38; p-JNK, anti-phosphorylated Jun N-terminal kinase; NFκB, nuclear factor kappa-light-chain-enhancer of activated B cells; AP-1, Activator protein 1; PPARs, Peroxisome proliferator-activated receptors; eCB, endocannabinoid; CB1, Cannabinoid receptor type 1; CB2, Cannabinoid receptor type 2; FAAH, Fatty acid amide hydrolase; CCL, C-C Motif Chemokine Ligand; WT, Wild-type; KO, Knock-out; IL-6, Interleukin 6; h, hours; SR1, SR141716A; SR2, SR144528; LDH-Abs, LDH activity; mRNA, messenger ribonucleic acid; AEA, anandamide; OEA, oleoylethanolamide; ERK1/2, Extracellular signal-regulated protein kinases 1 and 2; MAPK, Mitogen-activated protein kinase; TRPV1, transient receptor potential cation channel subfamily V member 1; WY, WY14643; TG, troglitazone; CPZ, capsazepine; YMT, Y-maze test; Ab, Ab25-35 peptide; VHI, vehicle; ScAb, Scrambled Ab25-35 peptide; PEA(3, 10, 30), PEA (3, 10, 30 mg/Kg); MWM, Morris Water Maze test; NORT, Novel Object Recognition test; MAP2, Microtubule-associated protein 2; GW6471, PPARα antagonist; BDNF, Brain-Derived Neurotrophic Factor; GDNF, Glial cell line-derived neurotrophic factor; nNOS, Neuronal nitric oxide synthase; AIF, Apoptosis-Inducing Factor; BACE1, Beta-secretase 1; APP, Amyloid precursor protein; WNT, Wingless and Int-1; pTau, phosphorylated Tau protein; pGSK3βB, phosphorylated-Glycogen synthase kinase 3-beta; Dkk1, Dickkopf WNT Signaling Pathway Inhibitor 1; DNA, Deoxyribonucleic acid; PARP1, Poly(ADP-Ribose)Polymerase1; C6, glial cell line; NO, Nitrogen Oxide; VEGF, Vascular endothelial growth factor; MMP-9, Matrix metallopeptidase 9; non-Tg, non-transgenic mouse model; 3xTg-AD, triple-transgenic mouse model of AD; MCAo, middle cerebral artery occlusion; Bax, Bcl-2 associated X-protein; Bcl-2, B-cell lymphoma 2; BrdU, Bromodeoxyuridine; MPTP, 1-methyl-4-phenyl-1,2,3,6-tetrahydropyridine; VaD, vascular dementia; OFT, Open-field test; PAR, Poly(ADP-ribose); μm, micrometer; TST, Tail suspension test; FST, Forced swim test; SPT, Sucrose preference test; p[Thr308]Akt, Phospho-Akt(Threonine308) Antibody; p[Ser9]Gsκ3β, Phospho-Gsκ3β(Serine9); p[Ser396]tau, Phospho-tau(Serine396); p[Ser536]p65, Phospho-p65(Serine536); IL-16, Interleukin 16; IL-5, Interleukin 5; MCSF, Macrophage colony-stimulating factor; MCP5, Murine Monocyte Chemoattractant Protein; IL-10, Interleukin 10; Glx, Glutamine/glutamate; NAA, N-acetylaspartate; GLT1, Glutamate Transporter-1; RI, recognition index; SNI, spare nerve injury; MPEP, 2-Methyl-6-(phenylethynyl) pyridine; MDCPG, (RS)-4-(1-amino-1-carboxyethyl)phthalic acid; eCB, endocannabinoid; AE, acylethanolamine; IPSI, ipsilateral; CONTRA, contralateral; 2-AG, 2-arachidonoylglycerol; NAAA, N-acylethanolamine acid amide hydrolase; Iba1, Ionized calcium-binding adaptor molecule 1; IκBα, nuclear factor of kappa light polypeptide gene enhancer in B-cells inhibitor alpha; NRF2, nuclear factor erythroid 2-related factor 2; MnSOD, Manganese superoxide dismutase; HO1, Heme oxygenase 1; T(1, 2, 3), time (1, 2, 3); Tg, transgenic; CX, cortex; HIPP, hippocampus; STR, striatum; CER, cerebellum; AAV, adeno-associated virus; Glu, glutamatergic neurons; AAV-Glu-FAAH, Animals overexpressing FAAH in glutamatergic neurons; PFC, prefrontal cortex; ROS, reactive oxygen species; CD11b, Integrin alpha M; STD, standard-diet group; HFD, high-fat diet; TBI, traumatic brain injury; DCX, doublecortine; d, days; NT3, neurotrophin 3; SAMP8, Senescence Accelerated Mouse-prone 8; SAMR1, Senescence-Accelerated Mouse-Resistant 1; SP, Stimulation protocol; α-syn, α-synuclein; TLR-4, Toll-like receptor 4; LPS, lipopolysaccharide; CFA, Complete Freund's Adjuvant; TG2, Tissue type 2 transglutaminase; TREM2, Triggering Receptor Expressed On Myeloid Cells 2; ARG1, Arginase 1. Bold font emphasizes significant results.
